# Objective validation of central sensitization in the rat UVB and heat rekindling model

**DOI:** 10.1002/j.1532-2149.2014.00469.x

**Published:** 2014-03-03

**Authors:** NS Weerasinghe, BM Lumb, R Apps, S Koutsikou, JC Murrell

**Affiliations:** School of Physiology and Pharmacology, University of BristolUK

## Abstract

**Background:**

The UVB and heat rekindling (UVB/HR) model shows potential as a translatable inflammatory pain model. However, the occurrence of central sensitization in this model, a fundamental mechanism underlying chronic pain, has been debated. Face, construct and predictive validity are key requisites of animal models; electromyogram (EMG) recordings were utilized to objectively demonstrate validity of the rat UVB/HR model.

**Methods:**

The UVB/HR model was induced on the heel of the hind paw under anaesthesia. Mechanical withdrawal thresholds (MWTs) were obtained from biceps femoris EMG responses to a gradually increasing pinch at the mid hind paw region under alfaxalone anaesthesia, 96 h after UVB irradiation. MWT was compared between UVB/HR and SHAM-treated rats (anaesthetic only). Underlying central mechanisms in the model were pharmacologically validated by MWT measurement following intrathecal *N*-methyl-d-aspartate (NMDA) receptor antagonist, MK-801, or saline.

**Results:**

Secondary hyperalgesia was confirmed by a significantly lower pre-drug MWT {mean [±standard error of the mean (SEM)]} in UVB/HR [56.3 (±2.1) g/mm^2^, *n* = 15] compared with SHAM-treated rats [69.3 (±2.9) g/mm^2^, *n* = 8], confirming face validity of the model. Predictive validity was demonstrated by the attenuation of secondary hyperalgesia by MK-801, where mean (±SEM) MWT was significantly higher [77.2 (±5.9) g/mm^2^ *n* = 7] in comparison with pre-drug [57.8 (±3.5) g/mm^2^ *n* = 7] and saline [57.0 (±3.2) g/mm^2^ *n* = 8] at peak drug effect. The occurrence of central sensitization confirmed construct validity of the UVB/HR model.

**Conclusions:**

This study used objective outcome measures of secondary hyperalgesia to validate the rat UVB/HR model as a translational model of inflammatory pain.

**What's already known about this topic?:**

- Most current animal chronic pain models lack translatability to human subjects.
- Primary hyperalgesia is an established feature of the UVB/heat rekindling inflammatory pain model in rodents and humans, but the presence of secondary hyperalgesia, a hallmark feature of central sensitization and thus chronic pain, is contentious.

**What does this study add?:**

- Secondary hyperalgesia was demonstrated in the rat UVB/heat rekindling model using an objective outcome measure (electromyogram), overcoming the subjective limitations of previous behavioural studies.

## 1. Introduction

The pursuit of novel analgesics to adequately treat chronic pain remains a pressing demand (Ballantyne et al., 2010), and progression in the development of mechanistically novel analgesics is remarkably low (Woolf, 2010). This has been attributed in part to mechanistic differences between the aetiopathogenesis of pain in animal models compared with the clinical pain conditions in man, highlighting the importance of translational models of chronic pain (Pound et al., 2004; Rice et al., 2008; Mogil et al., 2010).

A pragmatic approach of reverse translating a well-characterized human experimental pain model (Hoffmann and Schmelz, 1999; Benrath et al., 2001; Gustorff et al., 2004, 2013; Bishop et al., 2009) to animals (Saade et al., 2000, 2008; Davies et al., 2005; Themistocleous et al., 2006; Bishop et al., 2007) has been undertaken with an inflammatory pain model induced by ultraviolet (UV) irradiation in the UV-B range (UVB model). The UVB model is well characterized as a model of peripheral sensitization, where primary hyperalgesic responses peak between 24 and 48 h in rats and in humans.

In order for the UVB model to be utilized as a translational model of inflammatory pain, face, construct and predictive validity must be demonstrated in the animal model (Rice et al., 2008; Ballantyne et al., 2010). Therefore central sensitization, a fundamental underlying mechanism of chronic pain must also be evoked in the model (Woolf, 2007). Davies et al. (2011) provided behavioural evidence of central sensitization in the rat UVB model through induction of secondary hyperalgesia, which was enhanced and prolonged by heat rekindling (HR) of the irradiated area (UVB/HR model). A similar behavioural response is observed in humans following UVB/HR inflammation (Cookson et al., 2005), indicative of face validity. Despite this, central sensitization remains a controversial feature of the model due to conflicting evidence regarding the occurrence of secondary hyperalgesia using a similar protocol (Bishop et al., 2010). These differing results may be attributed to the use of behavioural outcome measures to detect secondary hyperalgesia, which introduces an element of subjectivity concerning judgment of withdrawal responses (Grigg et al., 2007). Despite the importance of demonstrating central sensitization in the UVB/HR model, it must also be acknowledged that evoking central sensitization is not sufficient evidence that the model mimics chronic pain, as some acute pain states can also recruit central pain mechanisms. This knowledge is also lacking for other experimental inflammatory pain models in animals that utilize algogenic substances such as carrageenan to induce inflammatory pain.

The current study aimed to demonstrate induction of central sensitization in the rat UVB/HR model, and thus present construct validity, using *objective* biomarkers of nociception (Willer, 1985). Biceps femoris electromyogram (EMG) withdrawal responses were measured following application of controlled mechanical pinch to the area of secondary hyperalgesia of the hind paw. A lower mechanical withdrawal threshold (MWT) was predicted in animals exposed to UVB/HR compared with SHAM-treated rats. MK-801, an *N*-methyl-d-aspartate (NMDA) receptor antagonist, was intrathecally (i.t.) administered to attenuate central sensitization. It was hypothesized MK-801 would increase the MWT to pinch in the area of secondary hyperalgesia in UVB/HR rats. This study provided evidence of central sensitization in the UVB/HR model through the induction of secondary hyperalgesia that was attenuated by intrathecal MK-801.

## 2. Materials and methods

### 2.1 Animals

All experiments were performed on adult male Wistar rats (weighing between 250 and 300 g; Harlan, Derby, UK) in accordance with the new European Union Directive and its predecessor the Animals Scientific Procedures Act. Rats were housed in groups of four in laboratory cages (480 × 375 × 210 mm) containing sawdust and *ad libitum* access to laboratory chow and water. Cages were enriched with a cylindrical tube. Ambient temperature was maintained between 19 and 23 °C. Animals were randomly divided into three treatment groups by randomly drawing coloured markers (three colours) from a container, with each colour representing a particular group (*n* = 8 per group, as determined from a power calculation using data derived from Davies et al., 2011, with an alpha value of 5% and beta value of 80%; Researcher's Toolkit, sample size calculator; Decision Support Systems, Fort Worth, TX, USA): SHAM treatment (anaesthetic only) + MK-801, UVB/HR + saline, UVB/HR + MK-801.

The UVB/HR model was induced using similar methodology to that of Davies et al. (2011) and described briefly below.

### 2.2 UVB irradiation

Animals were anaesthetized with a single intraperitoneal dose of sodium pentobarbital (pentobarbital sodium salt C-II; Sigma, St. Louis, MO, USA) (45 mg/kg) and were positioned in a UV chamber parallel to a UVB light source (TL01 tubes; Philips, Guildford, UK; *k*_max_ = 314 nm). UVB irradiation (290–320 nm wavelength) at a dose of 1000 mJ/cm^2^ was confined to the heel area of the right hind paw of the rat, with the remaining right hind paw shielded from exposure with black tape and the body with black cloth. The UVB source was calibrated prior to experiments using an irradiation probe placed at an equivalent distance from the light tubes as the animals' hind paw and the length of time needed to deliver an integrated dose of 1000 mJ/cm^2^ was determined. Rats in the SHAM treatment group were anaesthetized but not exposed to UVB.

### 2.3 Heat rekindling

HR was carried out 24 h after UVB treatment. Anaesthesia was induced as detailed above, and the previously irradiated heel area was heated at 45 °C using a feedback-controlled thermode (built in house) held firmly to the skin for a duration of 5 min. Rats in the SHAM treatment group were anaesthetized but did not receive HR treatment.

### 2.4 EMG recording

EMG experiments took place 4 days after initial UVB treatment.

#### 2.4.1 Surgical preparation

Animals were anaesthetized with halothane delivered by a face mask to allow cannulation of the jugular vein. Once venous access had been established, halothane administration was discontinued and anaesthesia maintained with a continuous intravenous infusion of alfaxalone (Alfaxan; Malvern Link, UK) at approximately 40 mg/kg/h titrated to maintain an adequate depth of anaesthesia for the experimental procedures. Depth of anaesthesia was monitored using a combination of clinical signs and respiratory rate. The trachea was cannulated to keep the airways patent and facilitate spontaneous respiration. A rectal probe was used to monitor body temperature, linked to a feedback-controlled thermal blanket to maintain body temperature between 37 and 38 °C.

Pharmacological agents were delivered i.t. via an implanted guide needle. For placement of the intrathecal guide needle, the rat was arched over a small beaker (placed beneath the pelvis) to aid visualization and to optimize the space between spinous processes in the lower lumbar region. The interspace between lumbar region L5 and L6 was determined to be an accessible region where hind paw afferents enter the spinal column and were identified as being roughly above the protrusion of the iliac crest. The stylet of a 22-gauge spinal needle was inserted into the space between the processes until a tail flick was elicited. A larger 21-gauge needle was then threaded over the stylet and secured in place using superglue. The stylet was left in place until time of injection in order to avoid obstruction of the lumen of the needle.

EMG responses in the biceps femoris to noxious stimulation of the right hind paw were used as an outcome measure of nociceptor activity. Muscle activity was recorded from two Teflon (Advent Research Materials, Oxford, UK)-coated stainless steel wires (approximately 10 cm) that were stripped to expose 2–3 mm of steel and threaded through a 25-gauge needle, with the stripped end bent over the tip to form a hook. The biceps femoris was pinched to make the muscle taut and the needle inserted 3–5 mm into the muscle belly. The needle was withdrawn slowly to allow the wire to remain embedded in the muscle. The second wire was placed around 1 cm from the first. A grounding earth wire was attached to a 25-gauge needle by a crocodile clip and inserted subcutaneously towards the neck of the rat. The signal across the wire electrode pair was amplified (×2000, NeuroLog preamplifier; Digitimer, Letchworth Garden City, UK), filtered (bandwidth 50 Hz to 5 kHz) and displayed on an oscilloscope (LeCroy 9304A, Quad 200 MHz oscilloscope; LeCroy, Chestnut Ridge, NY, USA). EMG was simultaneously captured at 5–10 kHz on a personal computer running Cambridge Electronic Design (CED) Spike 2 version 5.13, via a CED 1401*plus* [Cambridge Electronic Design (CED), Cambridge, UK].

At the end of the surgical preparation phase, the infusion rate of alfaxalone was reduced to 36 mg/kg/h and the plane of anaesthesia allowed to stabilize for an hour before EMG recording began. If no EMG response was detected in response to mechanical pinch up to 120 g/mm^2^, the anaesthetic infusion rate was further lowered and allowed to stabilize. The final infusion rate of alfaxalone ranged between 23 and 36 mg/kg/h for all experiments and varied randomly within group.

#### 2.4.2 Recording EMG response to a ramped mechanical stimulus

Mechanical withdrawal threshold (MWT) to a noxious mechanical stimulus applied to the right hind paw, adjacent to the UVB/HR area (and equivalent area in SHAM-treated rats) was recorded before and after drug administration to investigate the presence of secondary mechanical hyperalgesia.

A pinch of gradually increasing magnitude was delivered via a manually controlled pincher (built in house) to the mid region of the hind paw, between the footpads, until a biceps femoris EMG response was detected. A steady rate of application of the pinch was achieved by following a ramped guide trace with a rate of increase in pressure of 12 g/mm^2^/s. A number of different rates were trialled with 12 g/mm^2^ found to be an achievable and easily repeatable pinch that allowed not only precise measurement of MWT, but could also be discontinued quickly after an EMG response was detected, to avoid sensitization. MWT (g/mm^2^) was calculated on computer program Spike 2 version 5.13. The rate of application of the mechanical stimulus was kept constant to limit variability in withdrawal threshold, and the criteria for determining the EMG withdrawal threshold were standardized as being more than two standard deviation (SD) over background electrical activity of the muscle. An inter-stimulus interval of 15 min was maintained to avoid sensitization (Reeh et al., 1987; Carstens and Ansley, 1993). The pre-drug MWT (g/mm^2^) was calculated by averaging three consecutive pinches with a MWT range within 12.3 g/mm^2^, chosen from preliminary data (data not shown) as being one SD of pre-drug MWT in SHAM-treated rats (*n* = 5).

#### 2.4.3 Drug treatment

Once a pre-drug MWT was established, either saline (10 μL) or MK-801 (10 μg in 10 μL of saline) (Ascent Scientific, Cambridge, UK) was administered i.t. using a 25-μL fixed needle syringe (SGE Analytical Science, Victoria, Australia) and delivered via the previously placed guide needle (the stylet removed prior to administration). The dose of MK-801 used has previously been shown to attenuate central sensitization in other inflammatory models of pain, with no associated deficits in motor reflexes (Hama et al., 2003). Post-drug MWT was analysed every 15 min over a 60-min time course.

Prior to termination of the experiment, correct intrathecal placement of the needle was verified by monitoring the efficacy of intrathecal local anaesthetic (20 μL of 0.5% xylocaine; 0.5%, Astra Pharmaceuticals, Ltd., Kings Langley, UK) in abolishing the EMG withdrawal response to noxious pinch.

### 2.5 Statistical analysis

Statistical analyses were performed using GraphPad Prism version 5.03 (GraphPad Software, La Jolla, CA, USA). A one-way analysis of variance (ANOVA) followed by a Bonferroni multiple comparison *post hoc* test was used to assess differences between pre-drug MWT of all three groups. The absence of a significant difference between both of the UVB/HR treatment groups then allowed a pooling of UVB/HR pre-drug MWT values. A D'Agostino and Pearson omnibus normality test was performed, which established that the pooled UVB/HR data were normally distributed. An unpaired *t*-test compared whether pre-drug MWT significantly differed between the combined UVB/HR-treated rats and SHAM treatment. For post-drug analysis, pre-drug values were not pooled and a D'Agostino and Pearson omnibus normality test confirmed all groups were normally distributed. A two-way mixed-model ANOVA followed by Bonferroni *post hoc* test compared pre- and post-drug MWT within each group in a repeated measure design, and MWT between groups at various time points. Statistical significance was defined as *p* < 0.05.

## 3. Results

Rats received either UVB/HR or SHAM treatment (anaesthetic only) to the heel area of the right hind paw. Intrathecal saline or MK-801 was administered 4 days post-UVB treatment, where *n* = 8 per group: UV/HR + MK801, UVB/HR + saline, SHAM + MK-801.

### 3.1 UVB/HR causes secondary mechanical hyperalgesia

One rat in the UVB/HR (+MK-801) group had a pre-drug MWT greater than two SD above mean pre-drug MWT of all UVB/HR rats and was thus excluded from further analysis.

**Fig 1:**
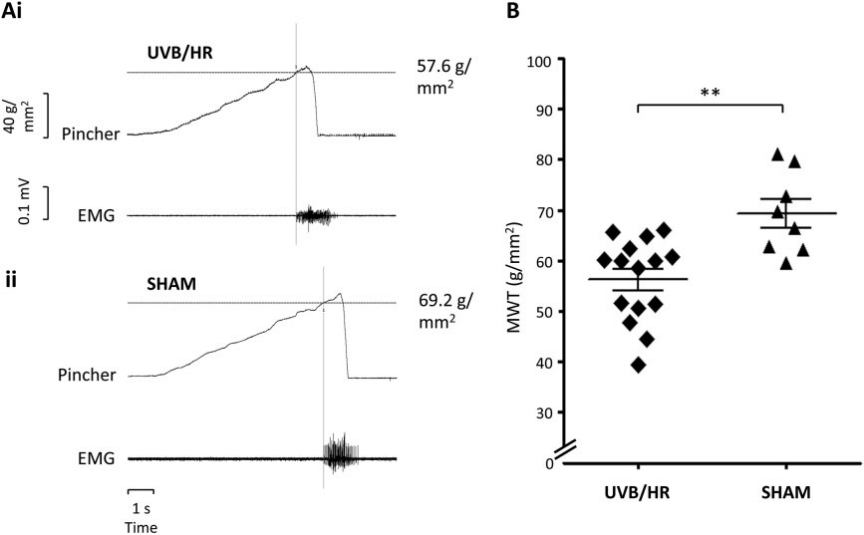
Figures showing pre-drug mechanical withdrawal threshold (MWT) to a pinch of increasing magnitude applied to the mid-region (i.e., area of secondary hyperalgesia) of the ipsilateral hind paw in UVB and heat rekindled (UVB/HR) and SHAM-treated rats. An example of raw data from a pre-drug recording is demonstrated in (Ai) from a UVB/HR rat and (Aii) from a SHAM-treated rat. Scale bars correlate to both (Ai) and (Aii). (B) Scatter plot showing overall pre-drug MWT in UVB/HR (*n* = 15) and SHAM-treated rats (*n* = 8). Each point represents data from an individual animal 4 days after initial UVB treatment or SHAM treatment. Horizontal lines represent mean ± standard error of the mean. **p < 0.01, unpaired *t*-test.

Mean [±standard error of the mean (SEM)] MWT was significantly lower (by 23%) in the area of secondary hyperalgesia for rats that received UVB/HR treatment [56.3 (±2.1) g/mm^2^, *n* = 15] compared with SHAM-treated rats [69.3 (±2.9) g/mm^2^ *n* = 8 *p* = 0.0015] (unpaired *t*-test, Fig. 1).

### 3.2 Secondary mechanical hyperalgesia in the UVB/HR model can be attenuated by MK-801

**Fig 2:**
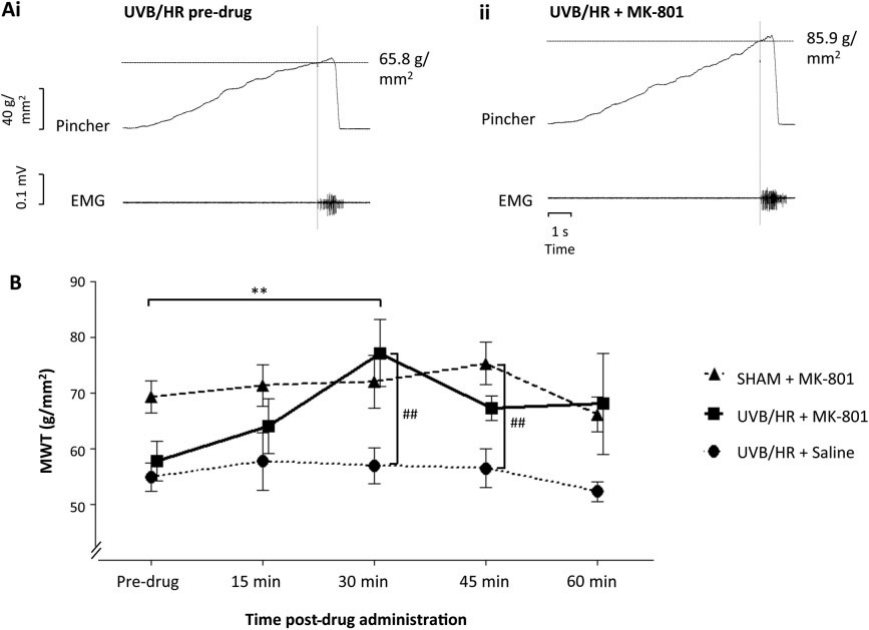
Figures showing the effect of intrathecal administration of *N*-methyl-d-aspartate receptor antagonist, MK-801 or saline on mechanical withdrawal threshold (MWT) in response to a pinch of increasing magnitude in the mid-region (i.e., area of secondary hyperalgesia) of the ipsilateral hind paw. An example of raw data from a UVB/HR rat is demonstrated in (Ai) showing a pre-drug MWT response and (Aii) MWT 30 min after MK-801 administration in the same rat. Scale bars correlate to both (Ai) and (Aii). (B) Time course of drug action in all groups [UVB/HR + MK-801 = 7 (squares), UVB/HR + saline =8 (circles), SHAM + MK-801 = 8 (triangles)] where MK-801 or saline was administered immediately after obtaining pre-drug responses. Post-drug responses were recorded every 15 min for 60 min, where each data point represents mean ± standard error of the mean 4 days after initial UVB or SHAM treatment. **p < 0.01 within group, ##*p* < 0.01 between groups, two-way analysis of variance following by Bonferroni *post hoc* test.

Centrally acting MK-801 increased MWT in UVB/HR rats in the area of secondary hyperalgesia, with a peak effect observed at 30 min post administration (Fig. 2A).

A two-way mixed-model ANOVA demonstrated a significant effect of time (*p* = 0.049) and group (*p* = 0.0032) but failed to show a significant interaction effect. A Bonferroni *post hoc* test determined a significant difference in time compared with pre-drug value at 30 min post-MK-801 administration in the UVB/HR group (*n* = 7). At this time point, MWT significantly increased by 33% compared with pre-drug MWT (Fig. 2B), where the mean (±SEM) MWT increased from 57.8 (±3.5) g/mm^2^ pre-drug to 77.2 (±5.9) g/mm^2^ 30 min post-MK-801 injection. There was no statistical difference at any other time point following MK-801 administration compared with pre-drug MWT in UVB/HR rats. MK-801 did not significantly alter MWT at any time point in the SHAM group (Fig. 2B), indicating that the modulation of MWT by MK-801 was due to alterations in the sensitized nociceptive pathway and not a general deficit in motor function alone. Saline had no effect on MWT in UVB/HR rats.

A Bonferroni *post hoc* test established a significant effect of group between UVB/HR + MK-801 and UVB/HR + saline (*n* = 8) at 30 min post-drug administration [77.2 (±5.9) g/mm^2^ compared with 57.0 (±3.2) g/mm^2^, respectively]. At this time point, mean MWT in the UVB/HR + MK-801 group was 35% higher than UVB/HR + saline. At 45 min post-drug, mean MWT in the SHAM + MK-801 group (*n* = 8) was significantly higher (33%) than UVB/HR + saline [75.3 (3.8) g/mm^2^ compared with 56.5 (±3.5) g/mm^2^]. There was no statistical difference between groups at any other time point.

## 4. Discussion

The UVB model is a model of inflammatory pain that overcomes the translational limitations of some established animal inflammatory pain models that cannot be readily applied to humans. A major component of construct validity in chronic pain models is the occurrence of central sensitization due to its importance as a key underlying mechanism in clinical chronic pain states. The occurrence of central sensitization in the UVB model has been debated in both rats and humans (Gustorff et al., 2004, 2013; Bishop et al., 2009, 2010; Andresen et al., 2011; Davies et al., 2011; Lorenzini et al., 2011, 2012), and the UVB model has primarily been utilized in human clinical trials to assess the efficacy of analgesics in modulating peripheral sensitization.

Davies et al. (2011) reported central sensitization for the first time in a rodent UVB model, which was further enhanced and prolonged upon the addition of HR (a 5-min application at 45 degrees) 24 h later (UVB/HR model), analogous to the extension of capsaicin-mediated hyperalgesia following the addition of HR in men (Dirks et al., 2003). HR has also been combined with UVB irradiation in human clinical trials where a 45-degree heat stimulus activates C-fibre nociceptors but can still be tolerated in men (Cookson et al., 2005; Wang et al., 2005). The increased magnitude and duration of secondary hyperalgesia in the UVB/HR model may be attributed to more sustained C-fibre nociceptor activity and therefore a more prolonged C-fibre input to the dorsal horn of the spinal cord compared with UVB exposure alone.

Here we aimed to demonstrate central sensitization in the UVB/HR model in rats using an objective biomarker of secondary hyperalgesia as an alternative to the behavioural measures used in previous studies evaluating the UVB model in rats.

### 4.1 Face validity

Face validity refers to whether the animal models possess similar characteristic responses to the human clinical condition. This was demonstrated by the reduction in MWT in the secondary area of UVB/HR rats in comparison with the corresponding site in rats that received SHAM treatment, which mimics behavioural responses reported upon model induction in humans (Cookson et al., 2005). Using EMG withdrawal thresholds to illustrate secondary hyperalgesia overcomes the limitations of evoked behavioural responses where there is an element of subjectivity in terms of what is deemed a withdrawal response and may be a potential source of disparity between the results from different studies (Bishop et al., 2010; Davies et al., 2011). However, it could be argued that using behavioural outcome measures in awake rats might better incorporate the multi-modal effects of pain due to engagement of the cerebral cortex.

A preliminary behavioural study established secondary hyperalgesia peaked at 96 h post-UVB irradiation when the UVB/HR model was induced under pentobarbital anaesthesia, and was thus the time point utilized in the current study. In the present study mechanical pinch rather than other modalities of noxious sensory stimulation was used to detect changes in withdrawal threshold due to the clinical relevance of mechanical secondary hyperalgesia in chronic pain states (Raja et al., 1984; Lamotte et al., 1991; Ali et al., 1996) and underlying mechanisms mainly thought to result from central sensitization (Lamotte et al., 1991; Treede et al., 1992; Klede et al., 2003).

### 4.2 Predictive validity

Predictive validity refers to the ability of an animal model to predict therapeutic efficacy in human clinical conditions and was previously demonstrated in the rat UVB/HR model using behavioural measures through attenuation of secondary hyperalgesia by systemic, but not a peripherally restricted topical dose of a non-steroidal anti-inflammatory drug (Davies et al., 2011). We also investigated predictive validity using intrathecal administration of a different class of analgesic drug, a non-competitive NMDA receptor antagonist, MK-801 (Latremoliere and Woolf, 2009). Antagonism of NMDA receptors by MK-801 has been shown to attenuate central sensitization in other inflammatory (Ren et al., 1992; Chen and Chen, 2000; Hama et al., 2003) and neuropathic pain models (Davar et al., 1991; Seltzer et al., 1991), and was therefore predicted to ameliorate secondary hyperalgesia in the UVB/HR model. At high doses MK-801 can potentially cause motor impairments (Hama et al., 2003) that may be unfavourable in analgesic drug studies that intend to measure sensory alterations alone. An absence of motor deficits is imperative in the present study due to the recording of reflex withdrawal responses. In order to control for any effects of MK-801 on motor function in our anesthetized preparation, we incorporated a SHAM group of rats that only underwent handling anaesthesia similar to the UVB/HR groups. MK-801 appeared to demonstrate an ‘over anti-hyperalgesic’ or analgesic effect (at 30 and 45 min post-MK-801 administration in the UVB/HR and SHAM groups, respectively, in comparison with UVB/HR + saline). However, MK-801 is unlikely to exert a significant analgesic effect or motor impairment as no change in MWT is observed in the SHAM group compared with pre-drug response. Also, the dose of MK-801 was chosen due to its ability to attenuate hyperalgesia in the absence of motor deficits (Hama et al., 2003). However, the study by Hama et al. (2003) was performed in awake behaving rats, and it is possible that our technique using an anaesthetized preparation is more sensitive in detecting subtle changes in motor responses. An alternative explanation may be that the surgical preparation adds an additional element of sensitization that is also diminished by MK-801. However, the most feasible justification for the subtle variation in results is attributed to the variability in EMG recordings to assess MWT between animals.

### 4.3 Construct validity

Construct validity refers to whether the induced pain condition in the animal model has similar underlying neurobiological mechanisms to the human clinical condition and was indicated by the occurrence of central sensitization, a key underlying mechanism of chronic pain, in the UVB/HR model. Central sensitization can be inferred through the lower MWT in UVB/HR compared with SHAM-treated rats in the area of secondary hyperalgesia, which was subsequently increased upon administration of the NMDA receptor antagonist MK-801.

The present study supports the findings of Davies et al. (2011), who postulated that previous conflicting results concerning the occurrence of secondary hyperalgesia in the rat UVB model may be due to differences in methodology used to detect secondary hyperalgesia between different studies.

The current study involved application of a ramped force to detect MWT, and due to the nature of EMG recordings, did not allow detection of the 50% withdrawal response, akin to the study by Bishop et al. (2010). An alternative reason for the disparity between our results and the findings of Bishop et al. (2009) may be the addition of HR to the UVB model, which increased the magnitude of central sensitization to allow detection of a change in 100% withdrawal threshold in the area of secondary hyperalgesia, which may not be detectable following UVB exposure only. Furthermore, the use of an anesthetized preparation compared with awake behaving animals removes conscious modulation of nociceptive withdrawal responses caused by attention and emotional state that may otherwise have led to variability in withdrawal responses. A potential disadvantage of studying withdrawal reflexes in anaesthetized animals is the potential for variability in the depth of anaesthesia between animals, which would inevitably affect the MWT (Grimby et al., 1966; Schouenborg and Kalliomaki, 1990; Boissonade and Matthews, 1993). This was controlled for in the present study by standardizing depth of anaesthesia as much as possible between animals by starting at a relatively high anaesthetic depth and decreasing the infusion rate of alfaxalone (and allowing time for stabilization) until an EMG response was observed to a standardized ramped mechanical pinch (up to 120 g/mm^2^). Furthermore, data were compared within animals over time in a repeated measures design in which all post-drug time points were compared with its pre-drug response.

The same experimenter induced the model and carried out the experiment, and was thus not blinded to the group or drug administered. This may be a potential limitation of the study; however, because an objective outcome measure was utilized, the non-blinded nature of the experiment was unlikely to confound data. One rat in the UVB/HR (+MK-801) group did not have evidence of secondary hyperalgesia pre-drug administration, which could be attributed to mechanical testing outside the boundary of secondary hyperalgesia. We did not map the area of secondary hyperalgesia prior to the experiment, which is a potential study limitation. However, it is difficult to behaviourally map the area of secondary hyperalgesia with any precision in an animal model. Other potential explanations for the failure to display secondary hyperalgesia may be due to stress, or adverse reactions to the anaesthetic or surgical preparation.

With any pain model, repeatability of the characteristics of the model is important. Following this study, we have utilized the UVB/HR model in other experiments with aims outside the scope of this paper and have consistently demonstrated secondary hyperalgesia.

## 5. Conclusion

A lower mechanical withdrawal threshold in UVB/HR compared with SHAM-treated rats in the secondary area confirms the development of central sensitization in the model, supported by attenuation of the observed secondary hyperalgesia by intrathecal MK-801 in the UVB/HR group only. An important feature of animal chronic inflammatory pain models is reliability in induction of detectable secondary hyperalgesia, which we have confirmed using a different methodology to others. These studies further validate the use of the UVB/HR model as a translational model of inflammatory pain, which may have utility in future pre-clinical rodent and human experimental analgesic drug development studies, and help limit drug redundancy between the pre-clinical and clinical interface.

### Author contributions

All authors contributed to the experimental design. Experimental work was carried out by N.S.W. All authors discussed the results and contributed to the writing of the manuscript.
